# Editorial: The role of birds in environmental transmission dynamics and impact on public health of zoonotic pathogens

**DOI:** 10.3389/fvets.2026.1825973

**Published:** 2026-04-01

**Authors:** Zorica Dakić, Duško Ćirović, Ivana Klun

**Affiliations:** 1 Department of Medical Microbiology, University Clinical Centre of Serbia, Belgrade, Serbia; 2 Faculty of Biology, University of Belgrade, Belgrade, Serbia; 3 Institute for Medical Research, University of Belgrade, Belgrade, Serbia

**Keywords:** avian influenza, *Chlamydia* (*Chlamydophila*) *psittaci*, One Health, public health risk, sentinels, surveillance, *Toxoplasma gondii*, West Nile virus

The abundance of zoonotic pathogens in the environment and the ease of their transmission has been a cause of concern and the focus of renewed research interests in the post-COVID era. Current research indicates that birds, both wild and domestic, can act as reservoirs for these pathogens, potentially leading to infections in other animals and humans. However, the asymptomatic nature of some of these infections often leads to an underestimation of the risk and impact of environmental contamination. This is further complicated by the complex life cycles of some of these pathogens, which can transition and survive through environmentally resistant stages, making it challenging to fully understand their transmission dynamics and identify reservoir hosts. Additionally, climate change is possibly altering the distribution of these pathogens by affecting bird habitats and migratory routes and thus assisting the species barrier crossing of bird pathogens with confirmed or suspected zoonotic potential. The primary aim of this Research Topic was to delve deeper into the intricate transmission dynamics of pathogens involving humans, birds, and the environment to explore the global epidemiological status of zoonotic pathogens with birds as confirmed or suspected reservoirs.

Interestingly, all contributions to the topic deal with obligatory intracellular pathogens, be they viruses, bacteria, or parasites. Most papers explored viral pathogens and the diseases they cause, particularly avian influenza (AI), which reflects its importance and the magnitude of the risk it may represent for global public health. In addition, avian coronaviruses, West Nile virus (WNV), as well as several virus families affecting wild birds whose zoonotic potential cannot be discounted are investigated. The topic includes two case reports of chlamydiosis, in human subjects and in parrots, respectively. Finaly, transmission of a protozoan parasite by birds in urban and rural environments is analyzed. Certainly, for all of these, but particularly for AI, a robust One Health approach is required to address the challenges of prevention and control (1). The global panzootic caused by the highly pathogenic avian influenza (HPAI) H5N1 virus affected more than 300 bird species (2). Since 2021, the virus has shifted from a seasonal threat to a year-round presence, spreading across Europe, Asia, Africa, and the Americas, and has recently reached the subantarctic region, causing widespread infections with high mortality rates and raising conservation concerns in vulnerable species of wild birds. In Europe, a record-high mortality was observed during the autumn migration in 2025. In addition to waterfowl, HPAI also strongly affects raptors, corvids, and seabirds. The virus also has the potential to mutate and spill over into mammals, including cattle and humans.

Showcasing the Research Topic is the paper by Montano Valle et al., who modeled the transmission of AI viruses at the human-animal-environment interface, specifically concerning the possibilities of spread from wild birds to poultry and pigs and then to humans in Cuba. The study utilized the One Health approach and geospatial modeling to determine the relative risk of AI virus transmission; the model included AI introduction and transmission risk factors, population density for animals as well as humans, the intensity of contact and a transmission parameter quantifying the relation between the number of infected and susceptible humans, and the number of infected animals of a certain production type. Identifying geographical areas at higher risk of AI virus transmission (especially due to interspecies spillover) would provide the health authorities with necessary information which would then facilitate adequate risk management through early warning and risk-based surveillance systems.

Several papers concentrated on the occurrence of a specific clade (2.3.4.4b) of the HPAI virus that was responsible for the recent panzootic surge, namely Cho et al. (in South Korea), Glazunova et al. (in the Russian Federation), and Ospina-Jimenez et al., who used epitope mapping to illuminate the origin, dissemination, and evolutionary dynamics of the virus in Latin America. Djurdjević et al. described the natural infection of (migratory) common cranes (*Grus grus*) with HPAI H5N1 in Europe (in Serbia). In addition to AI viruses, Khatun et al. explored the molecular biology of avian coronaviruses in environmental samples from migratory birds in Bangladesh. AI virus strains other than H5N1, such as A/H5 and A/H9, were also the subject of research in this country, and their association with biosecurity and hygiene practices in turkey farms was studied by Islam et al. Meng et al. presented the phylogenetic, pathogenicity, and transmission capacity analysis of a H9N2 strain, while Qu et al. examined the risk distribution of human infections with avian influenza A (H5N1, H5N6, H9N2, and H7N9) viruses in China.

Bourke et al. detected several virus families (Picobirnaviridae, Caliciviridae, Picornaviridae, and Sedoreoviridae) in U.S. mass-reared game-farm mallards, which highlighted spillover risk to naïve wild bird populations; they also raised the question of the plausible zoonotic potential of these viruses.

Birds are the reservoir hosts of the WNV, and the virus circulates in a mosquito-bird-mosquito transmission cycle, with humans being dead-end hosts. The WNV was detected in numerous (over 250) bird species, although corvids were particularly susceptible (3). Migratory birds are responsible for introducing WVN to new territories, where a transmission cycle is instigated when a vector mosquito species is present. Tamba et al. showed that the use of birds (Corvidae) as sentinels allows for the timely detection of WNV before the onset of human cases. Active surveillance was most effective when a minimum of 3.8 corvids per 100 km^2^ were tested, specifically between mid-June and mid-August.

Although the spread of viruses is explosive, zoonotic bacterial and parasitic pathogens, which may appear more discretely, should not be deemed less significant. The monitoring of zoonotic pathogens in 16 species of wild and captive birds was performed in the study of Softić et al., who detected only one case of WNV infection in a hooded crow in Sarajevo Canton, Bosnia and Herzegovina. However, *Chlamydia* (*Chlamydophila*) spp. was detected in numerous birds belonging to nine species, with *C. psittaci* identified in pigeons, both in urban feral and in domestically kept birds. AI, Chikungunya, and Usutu viruses were not detected. Detection of *C. psittaci* and WNV highlights the increased likelihood of zoonotic transmission, especially in densely populated urban environments.

Even though human psittacosis is considered a neglected disease, a recent spike of confirmed cases in several European countries which resulted in five deaths is alarming (4). The importance of timely diagnosis and treatment is underscored in the report by Shao et al., which describes a unique case of *C. psittaci* pneumonia followed by lower gastrointestinal ischemic necrosis, from which the patient eventually recovered. Since cases of human-to-human transmission are quite rare, birds are the principal reservoirs of infection. Birds, especially if they are asymptomatic carriers, also have great potential for spreading the disease internationally. Although the disease can be treatable, both in birds and humans, the role of veterinarians in its prevention cannot be stressed enough; quarantine and careful clinical and laboratory testing upon importation, as well as prophylactic antibiotics given in birdfeed in the weeks following, are a necessity. The absence of the above-mentioned practices may quickly result in serious disease or death in birds, as well as in the transmission of the disease from pet birds to their owners and other household members, which is clearly illustrated in the report by Horvatek Tomić et al..

The study by Penezić et al. explores the role of birds in the transmission of *Toxoplasma gondii*, the highest-ranked protozoan food-borne parasite in terms of the impact on public as well as individual health (5, 6). The highest molecular prevalence of *T. gondii* infection was in city-dwelling corvids, suggesting that these species may be good bioindicators for the parasite in cities, as well as in diurnal raptors, pheasants, and domestic (particularly rural backyard) chickens. Interestingly, it was shown that the environment birds lived in, rather than diet, had an influence on infection prevalence. In addition, the high occurrence of infection in domestic chickens and game birds also indicates a potential public health risk in case of inadequate meat preparation hygienic practices or insufficient cooking.

Avian species have a significant role in various pathogen transmission chains, principally due to opportunities provided by proximity between domestic, wild, and migratory birds, as well as other mammal species including humans, which facilitate pathogen spread. Birds traverse international and geographic borders and thus contribute to pathogen introduction to naïve and/or remote territories. The emergence and re-emergence of diverse zoonotic diseases, often originating from wildlife, along with their intensified geographical dissemination, represents one of the greatest global health challenges in the current century, so it is imperative adequate surveillance systems are in place. Equally important is the reliance on the One Health approach to achieve control of these diseases and, crucially, to prevent their transmission at both the local and global levels.
